# Effects of Caffeine Supplementation on Exercise Performance in Volleyball Players: A Systematic Review and Meta-Analysis

**DOI:** 10.3390/nu17101709

**Published:** 2025-05-18

**Authors:** Bin Chen, Chuanmin Zhang, Zhenghong Xu, Yiqian Li, Li Guo, Yinhang Cao, Olivier Girard

**Affiliations:** 1 Department of Public Physical Education, Fujian Agriculture and Forestry University, Fuzhou 350002, China; chenbin@fafu.edu.cn (B.C.); 13809504416@163.com (Z.X.); 2 School of Physical Education, Tianjin University of Sport, Tianjin 300060, China; zcmtjus@126.com; 3 School of Athletic Performance, Shanghai University of Sport, Shanghai 200438, China; 2421811007@sus.edu.cn; 4 School of Exercise and Health, Shanghai University of Sport, Shanghai 200438, China; guoli@sus.edu.cn; 5 School of Human Sciences (Exercise and Sport Science), The University of Western Australia, Perth 6009, Australia; oliv.girard@gmail.com

**Keywords:** ergogenic aid, sports performance, physical performance, team sports

## Abstract

**Background/Objectives:** The ergogenic effects of caffeine in team sports, particularly volleyball, have received significant research attention. This study sought to examine the effects of caffeine on both volleyball-specific and general performance outcomes. **Methods:** This systematic review comprises 11 studies, each utilizing a blinded crossover experimental design. A meta-analysis was conducted using a random-effect model to determine the standardized mean difference (SMD), estimated by Hedges’ g, with a 95% confidence interval (CI). **Results:** Caffeine supplementation improved volleyball-specific outcomes, including attack and serve accuracy (SMD: 0.50; 95% CI: 0.11–0.90; *p* = 0.01). Regarding nonspecific outcomes, caffeine increased single-jump performance (SMD: 0.23; 95% CI: 0.02–0.44; *p* = 0.03), repeated-jump performance (SMD: 0.51; 95% CI: 0.05–0.96; *p* = 0.03), and handgrip strength (SMD: 0.23; 95% CI: 0.03–0.42; *p* = 0.02), while decreasing agility test completion time (SMD: −0.32; 95% CI: −0.60–0.03; *p* = 0.03). Furthermore, caffeine increased the frequency of positive game actions during simulated volleyball matches (SMD: 0.84; 95% CI: 0.26–1.43; *p* < 0.01). **Conclusions:** Caffeine supplementation enhances physical performance and volleyball-specific actions during competition, supporting its role as an effective ergogenic aid for volleyball players.

## 1. Introduction

Volleyball is one of the most popular team sports globally [1]. During matches, players must execute sport-specific technical actions, such as spiking, serving, and blocking, along with rapid movements including acceleration, deceleration, and directional changes, often over extended periods (three to five sets; 1–3 h) [2,3,4]. Thus, maintaining high physical performance while executing these volleyball-specific skills is crucial for competitive success.

Caffeine is a widely used ergogenic aid among athletes across various sports [5,6,7] and is classified as a safe supplement by the International Society of Sprots Nutrition (ISSN) [8]. Caffeine popularity has risen due to its positive effect on both aerobic and anaerobic activities [9,10]. It increases strength and power by promoting intracellular Ca^2+^ release and Na^+^/K^+^-ATPase pump activity [11,12], while also delaying fatigue onset by activating the central nervous system and blocking the adenosine receptors [13,14]. The benefits of caffeine supplementation in individual sports such as running or cycling are well established [9,10,15,16]. However, there is limited information on its ergogenic effects in team sports, which require a combination of physical and sport-specific technical and tactical skills.

A meta-analysis of Salinero et al. [17] demonstrated the effectiveness of caffeine on physical performance (e.g., total running distance, number of sprints) in various team sports (e.g., soccer, rugby and American football), but did not examine its effects on sport-specific skills. Building on this, a recent meta-analysis conducted by Diaz-Lara et al. [18] reported that caffeine supplementation enhances sport-specific actions (e.g., sprint frequency, body impacts) in intermittent sports such as team, racket, and combat sports. However, their meta-analysis lacked a focused evaluation of sport-specific skills in team sports players and included only three volleyball-specific studies, which limits its relevance to volleyball performance.

As a growing number of studies focus on how caffeine affects volleyball performance, it is important to give this area individual attention rather than inferring results from a range of team sports with differing physical and skill demands. A comprehensive systematic review with meta-analysis specifically targeting volleyball would provide valuable insights for scientists, coaches, and athletes interested in understanding the effects of caffeine in this sport. Therefore, the aim of this study was to enhance knowledge on the impact of caffeine supplementation on volleyball players by exploring its effects on both volleyball-specific and general performance outcomes.

## 2. Materials and Methods

### 2.1. Literature Search

For this systematic review with meta-analysis, we followed the Preferred Reporting Items for Systematic Reviews and Meta-Analyses 2020 guidelines [19]. This systematic review has been registered in the International Prospective Register for Systematic Reviews (PROSPERO; registration number: CRD42024583602). Search terms included a mix of Medical Subject Headings (MeSH) and free-text words for key concepts related to caffeine and volleyball. Articles were systematically identified using the following search syntax: (concept 1) (caffeine OR coffee) AND (concept 2) (supplement OR supplementation OR ergogenic aid) AND (concept 3) (volleyball OR team sports OR simulated sports) AND (concept 4) (performance OR athletic performance OR sports performance OR physical performance). This search syntax was applied in 4 different databases, PubMed, Embase, Web of Science, and Scopus. The literature search was performed by two separate authors (B.C., C.Z.) and conducted through March 2025.

### 2.2. Study Selection

Article selection followed the participants, interventions, comparators, outcomes, study design (PICOS) framework [20]. This systematic review only incorporated studies with crossover experimental designs in which the ingestion of caffeine was compared with a placebo in a single- or double-blind randomized manner and outcomes were associated with exercise performance in volleyball players. We considered all forms of caffeine supplementation (i.e., capsules, chewing gum, powder, and caffeinated beverages), provided the effect of caffeine could be isolated. Inclusion criteria are detailed in Table 1.

### 2.3. Data Extraction

The following information was extracted from each study: (1) first author and publication year; (2) participant characteristics (sample size, age, sex, sports performance level, and habitual caffeine intake); (3) caffeine supplementation method, timing, and dosage; (4) exercise protocol; and (5) main findings.

Performance outcomes were categorized as follows:

(a) Volleyball-specific tests [21,22]: spike-ball velocity (standing and jumping); attacking and serving accuracy; and block and attack jumps.
(b) Nonspecific tests [23]: single and repeated jumps; handgrip strength; and agility.
(c) Game actions during real or simulated competition [18]: frequencies of positive, neutral, and negative game actions.

### 2.4. Assessment of Methodological Quality

The methodological quality of the included studies was assessed using the Cochrane risk-of-bias tool for randomized controlled trials following the Cochrane Collaboration Guidelines [24]. This tool evaluates seven bias domains: random sequence generation (selection bias), blinding of participants and personnel (performance bias), blinding of outcome assessment (detection bias), incomplete outcome data (attrition bias), selective reporting (reporting bias), and other potential sources of bias (other bias).

### 2.5. Statistical Analyses

This meta-analysis compared the effects of caffeine versus placebo on performance outcomes using the standardized mean difference (SMD) with 95% confidence intervals (CI). For each outcome, the SMD was calculated using the mean and standard deviation values from placebo and caffeine trials, along with the sample sizes and correlations between trials. Heterogeneity was assessed using the *I*^2^ statistic and interpreted as follows: low (*I*^2^ < 25%), moderate (25% ≤ *I*^2^ ≤ 50%), and high (*I*^2^ > 50%) [16]. All meta-analyses were conducted using a random-effect model. Data analyses were performed using Review Manager (RevMan, Version 5.4.1; Cochrane, London, UK). Sensitivity analyses, funnel plots, and meta-regression were conducted using Stata software (Stata Corp, Version 15.0, College Station, TX, USA). Statistical significance was set at *p* < 0.05.

## 3. Results

### 3.1. Study Characteristics

**Figure 1:**
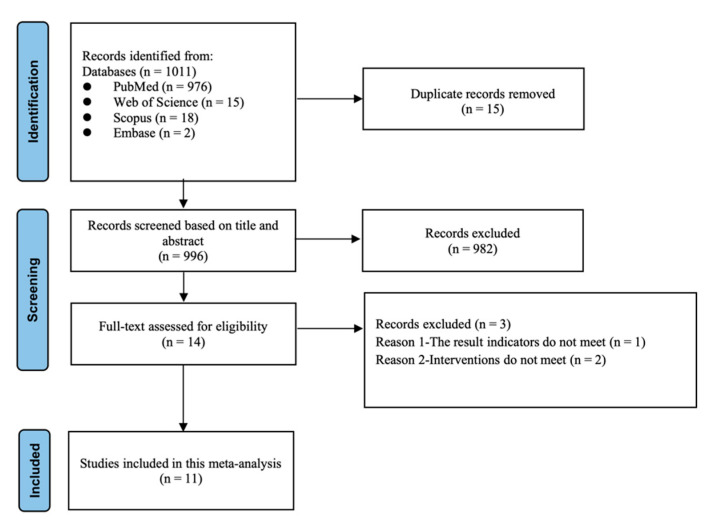
PRISMA flow diagram showing study selection.

A total of 1011 studies were initially identified. After removing duplicates, titles and abstracts were screened (Figure 1). Eleven studies, comprising 68 effect sizes, met the eligibility criteria and were selected for quantitative analysis [25,26,27,28,29,30,31,32,33,34,35].

Table 2 presents the characteristics of the included studies, which involved 137 volleyball players (49 men, 88 women). Based on the performance classification by McKay et al. [36], 59 participants (29 women) were categorized as highly trained/national-level (tiers 3) and 78 (59 women) as elite/international-level (tiers 4) players. Caffeine doses ranged from 1 to 6 mg/kg and were administered through capsule (four studies), energy drink (three studies), gum (two studies), powder (one study), and power bar (one study). Most studies administered caffeine 60 min prior to testing. Exceptions included Fernandez et al. [35], who administered it 30 min prior, Kaszuba et al. [30] and Filip-Stachnik et al. [28], who provided it 15 min before, and Pfeifer et al. [26], who gave caffeine “immediately prior to and during the competition”. Seven studies reported caffeine habituation, with most participants classified as low caffeine consumers (i.e., 30–100 mg/d) [37].

Eleven studies reported nonspecific physical performance, five focused on volleyball-specific skills [25,28,30,31,34], and two conducted tests during a simulated volleyball match [25,34].

### 3.2. Methodological Quality

**Figure 2:**
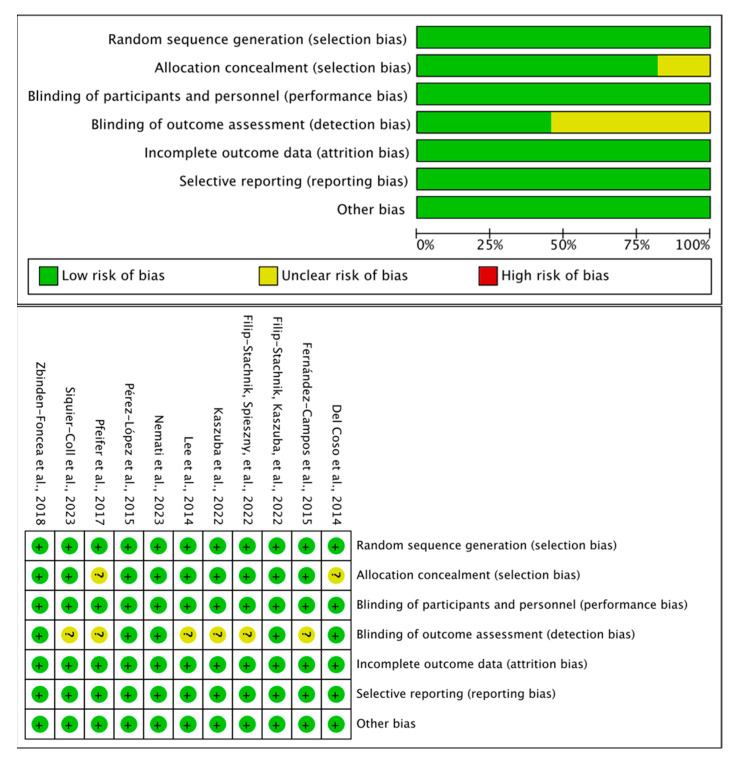
Risk-of-bias summary for the included studies [25,26,27,28,29,30,31,32,33,34,35].

The quality and risk-of-bias assessment suggested that all 11 studies had a low risk of bias in “random sequence generation,” “blinding of participants and personnel,” “incomplete outcome data,” “selective reporting,” and “other bias.” However, two studies demonstrated an unclear risk in “allocation concealment.” Additionally, six studies had an unclear risk regarding “blinding of outcome assessment” due to insufficient details (Figure 2).

### 3.3. Meta-Analysis Results

#### 3.3.1. Volleyball-Specific Outcomes

**Figure 3:**
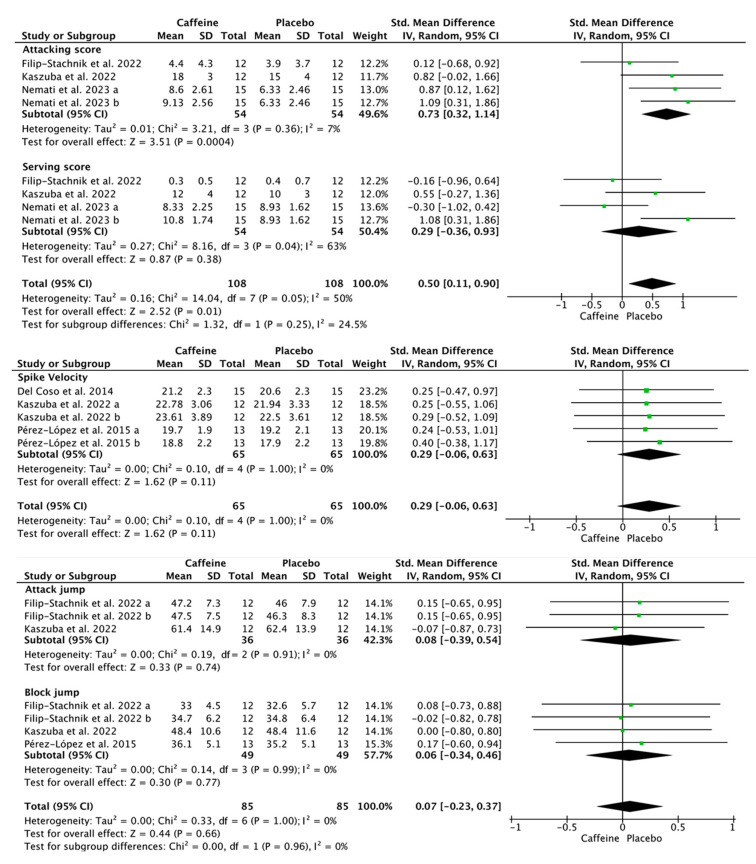
Effects of caffeine on volleyball-specific skills including attacking and serving accuracy, spike velocity, and attack and block jump height. Green squares represent the study-specific estimate, while diamonds indicate the pooled estimate from the random-effect model. CI: confidence interval, SD: standard deviation [25,28,30,31,34]. “a”, “b” represents the number of trials of the same study.

Compared with placebo, caffeine supplementation enhanced overall accuracy in attacking and serving tests (SMD = 0.50; 95% CI = 0.11–0.90; *p* < 0.05; *I*^2^ = 50%). In the subgroup meta-analysis, caffeine improved attacking accuracy (SMD = 0.73; 95% CI = 0.32–1.14; *p* = 0.0004; *I*^2^ = 7%; four effect sizes from two studies), but not serving accuracy (SMD = 0.29; 95% CI = −0.36–0.93; *p* = 0.38; *I*^2^ = 63%; four effect sizes from two studies) (Figure 3). Furthermore, caffeine had no effect on spike velocity (SMD = 0.29; 95% CI = −0.06–0.63; *p* = 0.11; *I*^2^ = 0%; five effect sizes from three studies), block jump height (SMD = 0.06; 95% CI = −0.34–0.46; *p* = 0.77; *I*^2^ = 0%; four effect sizes from three studies), or attack jump (SMD = 0.08; 95% CI = −0.39–0.54; *p* = 0.74; *I*^2^ = 0%; three effect sizes from two studies) (Figure 3).

#### 3.3.2. Nonspecific Outcomes

**Figure 4:**
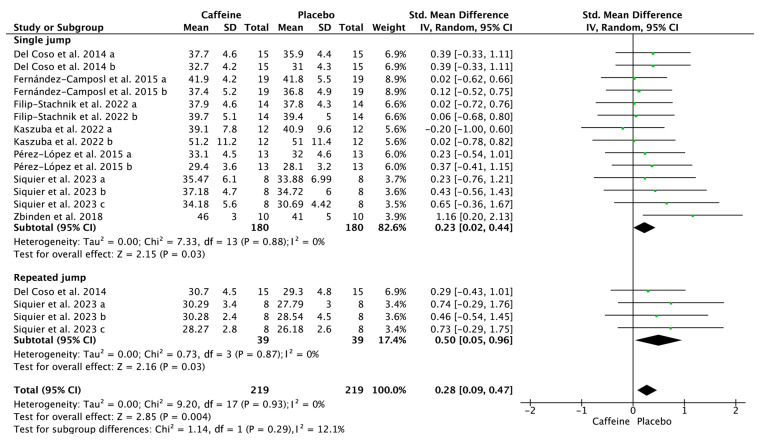
Effects of caffeine on single- and repeated-jump performance. Green squares represent the study-specific estimate, while diamonds indicate the pooled estimate from the random-effect model. CI: confidence interval; SD: standard deviation [25,27,29,30,32,34,35]. “a”, “b”, “c” represents the number of trials of the same study.

**Figure 5:**
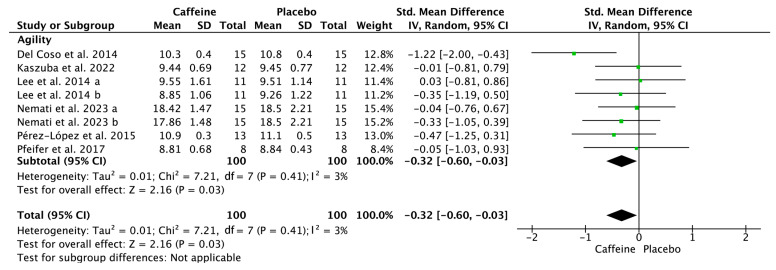
Effects of caffeine on agility. Green squares represent the study-specific estimate, while diamonds indicate the pooled estimate from the random-effect model. CI: confidence interval, SD: standard deviation [25,26,30,31,33,34]. “a”, “b” represents the number of trials of the same study.

**Figure 6:**
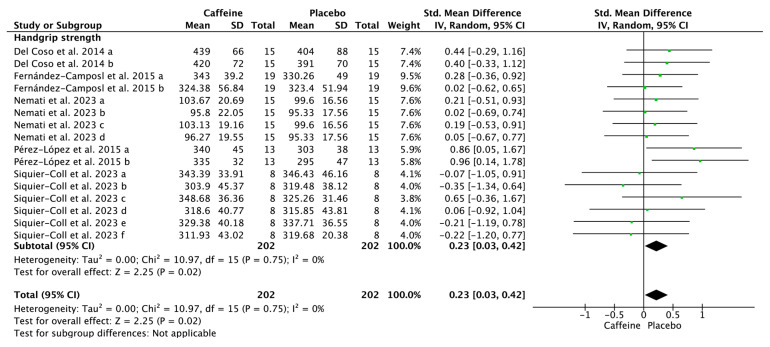
Effects of caffeine on handgrip strength. Green squares represent the study-specific estimate, while diamonds indicate the pooled estimate from the random-effect model. CI: confidence interval, SD: standard deviation [25,31,32,34,35]. “a”, “b”, “c”, “d”, “e”, “f” represents the number of trials of the same study.

Figure 4, Figure 5 and Figure 6 depict the effects of caffeine on non-volleyball specific physical performance. Caffeine supplementation enhanced the height of a single jump (SMD = 0.23; 95% CI = 0.02–0.44; *p* = 0.03; *I*^2^ = 0%) and repeated jumps (SMD = 0.50; 95% CI = 0.05–0.96; *p* = 0.03; *I*^2^ = 0%) (Figure 4). Furthermore, it shortened agility test completion time (SMD = −0.32; 95% CI = −0.60–−0.03; *p* = 0.03; *I*^2^ = 3%) (Figure 5) and enhanced handgrip strength (SMD = 0.23; 95% CI = 0.03–0.42; *p* = 0.02; *I*^2^ = 0%) (Figure 6).

#### 3.3.3. Outcomes During Simulated Volleyball Competition

**Figure 7:**
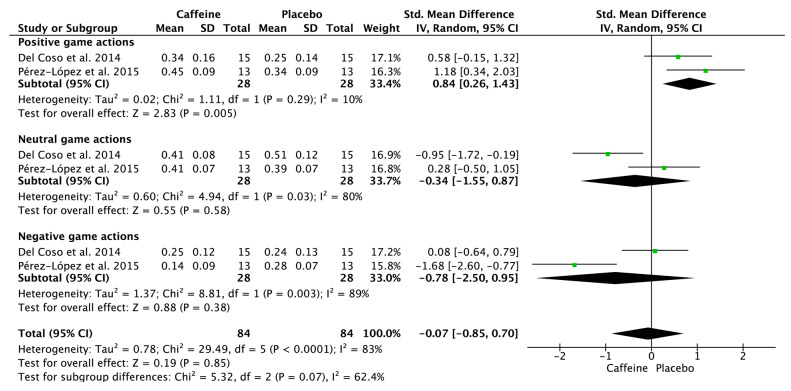
Effects of caffeine on positive, neutral, and negative game actions. Green squares represent the study-specific estimate, while diamonds indicate the pooled estimate from the random-effect model. CI: confidence interval, SD: standard deviation [25,34].

Meta-analyses of volleyball-specific actions during real or simulated competition indicated that caffeine supplementation significantly increased positive game actions compared with placebo (SMD = 0.84; 95% CI = 0.26–1.43; *p* < 0.01; *I*^2^ = 10%; two effect sizes from two studies) (Figure 7). However, caffeine had no significant effect on negative (SMD = −0.78; 95% CI = −2.50–0.95; *p* = 0.38; *I*^2^ = 89%) or neutral game actions (SMD = −0.34; 95% CI = −1.55–0.87; *p* = 0.58; *I*^2^ = 80%) (Figure 7).

## 4. Discussion

### 4.1. Key Findings

The novelty of our meta-analysis lies in its focused examination of caffeine’s effects on both physical performance and sport-specific skills in volleyball players. Our results demonstrate that caffeine supplementation (3–6 mg/kg) significantly enhances volleyball-specific abilities, such as attacking and serving accuracy, as well as general performance metrics, including vertical jump height, reaction time, and handgrip strength. Furthermore, caffeine increased positive game actions during simulated volleyball matches. Overall, caffeine supplementation may be a valuable intervention for volleyball players seeking acute performance enhancement.

### 4.2. Effects of Caffeine Supplementation on Volleyball-Specific and Nonspecific Performance

Jumping ability is a pivotal skill in volleyball, particularly for actions including blocking, serving, and spiking [38]. During a five-set match, elite volleyball players execute between 250 and 300 high-intensity movements, with jumps accounting for approximately 50%–60% of these actions [39]. The present meta-analysis found that caffeine supplementation does not significantly affect the height achieved in volleyball-specific jumps, such as attack and block jumps (Figure 3), but did improve both single- and repeated-jump performance (Figure 4). This discrepancy may stem from differences in caffeine dosage and habitual intake. Studies on single- and repeated-jump performance primarily used caffeine doses ranging from 3 to 6 mg/kg [27,28,29,32,34,35]. In contrast, studies on volleyball-specific jumping performance, such as attack and block jumps, predominantly employed a 3 mg/kg dose [25,30]. Of the three studies examining attack and block jumps, only the one using 6 mg/kg reported a significant increase in attack jump height [28]. The two studies that used 3 mg/kg found no significant improvement [25,30]. Supporting this, Nemati et al. [31] found 6 mg/kg more effective than 3 mg/kg in enhancing lower-body muscle strength, potentially due to increased Ca^2+^ release and improved muscle contractility at higher doses [40]. Additionally, participants in volleyball-specific jumping studies were typically mild-to-moderate caffeine consumers (181–231 mg/day), while those in single- and repeated-jump studies were generally light or caffeine-naïve consumers (<60 mg/day). Chronic caffeine intake can upregulate adenosine receptors, which may diminish caffeine’s ergogenic effects over time [41]. Similarly, Sökmen et al. [42] suggested that de-habituation from caffeine or higher caffeine doses may enhance performance for habitual users in team sports. Taken together, these findings suggest that higher doses (>3 mg/kg) may be necessary to enhance attack and block jump performance in habitual caffeine users, though this hypothesis warrants further investigation.

While caffeine supplementation did not influence block jump height, attack jump height, or spiking velocity, it significantly enhanced both attacking and serving accuracy (Figure 3). These improvements may be attributed, at least in part, to its neurocognitive effects [31]. A review of double-blind, placebo-controlled studies has demonstrated that caffeine enhances both simple and complex attention tasks, as well as the functioning of alerting and executive control networks [43]. Caffeine acutely improves neural network efficiency in the human cerebral cortex, which may enhance cognitive functions relevant to performance [44]. These cognitive benefits can translate into better performance in tasks requiring focus, concentration, and decision-making [45]. From a physiological perspective, caffeine’s effects on central and peripheral mechanisms vary with dosage. At higher doses, it increases Ca^2+^ release from the sarcoplasmic reticulum, which enhances skeletal muscle contractility, while lower doses primarily exert effects through adenosine receptor antagonism [46]. While moderate doses (e.g., 6 mg/kg) may not significantly improve strength outcomes in exercises such as the bench press or squat, higher doses (e.g., 8 mg/kg) show more pronounced effects on maximal strength compared to placebo [47]. These findings suggest that higher caffeine doses may be required to significantly enhance block and attack jump height and spike velocity, which rely heavily on muscle strength and power.

Although caffeine supplementation did not significantly affect spiking velocity, it significantly enhanced handgrip strength (Figure 6). This difference may be due to the handgrip strength test primarily engaging smaller muscle groups of the hand and forearm, which are more susceptible to fatigue and caffeine sensitivity [48,49]. In contrast, spiking encompasses complex whole-body coordination, involving multiple large muscle groups, including the lower limbs such as the gluteus maximus, quadriceps femoris, hamstrings, and gastrocnemius for jumping and force production; core muscles including the rectus abdominis, erector spinae, and external obliques for stability and force transfer; and upper limbs and shoulder muscles such as the pectoralis major, anterior, middle, and posterior deltoid, trapezius, and triceps brachii for striking [50,51]. The response to caffeine in larger muscle groups tends to be less pronounced than in smaller ones, consistent with previous results that caffeine supplementation enhances upper-body but not lower-body strength [15].

Beyond jump ability and handgrip strength, caffeine supplementation also enhances other performance attributes, such as agility (Figure 5), which are crucial in volleyball. This effect can be attributed to the small playing area, position-specific roles, and short rally durations in volleyball, all of which necessitate rapid deceleration and accelerations and explosive movements [52]. Additionally, caffeine’s ability to improve alertness and wakefulness likely contributes to faster reaction times [45].

### 4.3. Effects of Caffeine Supplementation on Game Actions During Volleyball Competition

Caffeine supplementation enhanced positive game actions during simulated volleyball competition, but did not significantly affect neutral or negative game actions (Figure 7). These findings align with a previous meta-analysis on the acute effects of caffeine in intermittent sports (e.g., team, racket, and combat sports) that which require decision-making and high-intensity efforts during real or simulated competitions [18]. Caffeine supplementation increased high-intensity, sport-specific actions and improved success rates, but did not significantly lower the frequency of neutral or negative actions [18]. Hence, caffeine enhances physical performance that translates into improved sports performance, primarily improving positive game actions rather than reducing neutral or negative ones, particularly in team sports. However, since the included studies used simulated volleyball competitions, it remains unclear whether similar effects would be observed in real competitive settings. Future research should focus on quantifying the effects of caffeine during actual volleyball matches to better reflect the demands of official play.

#### Limitations

Despite its strengths, this study has several limitations. First, some outcomes in the meta-analysis were based on a small number of studies (*n* = 3). Second, while only double-blinded, randomized controlled trials were included, variations in caffeine supplementation methods across studies make it difficult to determine the optimal method for maximizing caffeine’s benefits on volleyball-specific actions during competition. Third, different caffeine sources (e.g., capsule, gum, drink, powder) may have influenced caffeine pharmacokinetics [53], potentially affecting study outcomes. In addition, variations in athletes’ training levels could have modulated the ergogenic response to caffeine [54], but the limited number of studies precluded subgroup analysis based on competition level. Finally, the current evidence does not allow us to explore the potential influence of sex on caffeine’s ergogenic effects, a factor previously noted as relevant [55]. Future research is needed to confirm the effects of caffeine supplementation on volleyball-specific performance and determine whether these effects vary based on training status, sex, age, or method of caffeine delivery.

## 5. Conclusions

Pre-exercise caffeine supplementation enhanced both volleyball-specific performance (i.e., attack and serve accuracy) and non-volleyball-specific performance (i.e., vertical jump height, agility, and handgrip strength). Furthermore, a moderate caffeine dose increased the frequency of positive game actions during simulated volleyball competition. These findings indicate that caffeine supplementation boosts physical performance and likely improves volleyball-specific execution in competitive settings.

## Figures and Tables

**Table 1:** PICOS criteria for the inclusion of studies in the systematic review.

Parameter	Inclusion Criteria
Participant	Volleyball players aged ≥ 18 years
Intervention	Caffeine supplementation (i.e., capsules, gum, powder, and caffeinated beverages), provided the effect of caffeine could be isolated.
Comparator	Placebo supplementation
Outcomes	Volleyball-specific tests: spike-ball velocity (standing and jumping); attacking and serving accuracy; and block and attack jumps. Nonspecific tests: single and repeated jumps; handgrip strength; and agility. Game actions during real or simulated competition: frequencies of positive; neutral; and negative game actions.
Study Design	Randomized single- or double-blind crossover designs. Only studies that were published in English and as original research (i.e., not a conference abstract or review) were included.

**Table 2:** General characteristics of included studies.

Study	Sample + Age (years) + Level	Habitual Caffeine Intake (mg/day)	Caffeine Form + Does (mg/kg or mg) + Timing (min)	Comparator	Exercise Protocol	Main Findings
Lee et al. (2014) [33]	11 F; 21 ± 1; Elite	75	Capsule; 6; 60	Placebo (cellulose)	AG test + RSE (5 × 4 s)	→AG: (Caffeine: 8.9 ± 1.1 vs. Placebo: 9.3 ± 1.2)
Del Coso et al. (2014) [34]	15 M; 22 ± 6; Highly Trained	30	Energy drink; ~3 (239); 60	Placebo (decaffeinated drink)	VS + CMJ + SJ + RJ + AG+ HS + SM test	↑VS: +2.9% (21.2 ± 2.3 vs. 20.6 ± 2.3); Positive game actions: +36.0% (0.3 ± 0.2 vs. 0.3 ± 0.1); Negative game actions: +4.2% (0.3 ± 0.1 vs. 0.2 ± 0.1); CMJ: +5.0% (37.7 ± 4.6 vs. 35.9 ± 4.4); SJ: +5.5% (32.7 ± 4.2 vs. 31 ± 4.3); RJ: +4.8% (30.7 ± 4.5 vs. 29.3 ± 4.8); HS: +8.7% (439.0 ± 66.0 vs. 404.0 ± 88.0) ↓Positive game actions: −19.6% (0.4 ± 0.1 vs. 0.5 ± 0.1); AG: −4.6% (10.3 ± 0.4 vs. 10.8 ± 0.4)
Fernández et al. (2015) [35]	19 F; 22 ± 4; Elite	N.A.	Energy drink; 2; 30	Placebo (decaffeinated drink)	CMJ + SJ + HS test	→CMJ: (41.9 ± 4.2 vs. 41.8 ± 5.5); SJ: (37.4 ± 5.2 vs. 36.8 ± 4.9); HS: (324.4 ± 56.8 vs. 323.4 ± 51.9). ↑HS: +3.9% (343.0 ± 39.2 vs. 330.3 ± 49.0)
Pérez-López et al. (2015) [25]	13 F; 25 ± 4; Highly Trained	N.A.	Energy drink; 3; 60	Placebo (decaffeinated drink)	VS + SM test	↑Spike ball velocity: +5.0% (18.8 ± 2.2 vs. 17.9 ± 2.2); Block jump height: +2.6% (36.1 ± 5.1 vs. 35.2 ± 5.1); Positive game actions: +32.4% (0.5 ± 0.1 vs. 0.3 ± 0.1); CMJ: +3.4% (33.1 ± 4.5 vs. 32 ± 4.6); SJ: +4.6% (29.4 ± 3.6 vs. 28.1 ± 3.2); HS: +13.6% (335.0 ± 32.0 vs. 295.0 ± 47.0) ↓Negative game actions: −50.0% (0.1 ± 0.1 vs. 0.3 ± 0.1); AG: −1.8% (10.9 ± 0.3 vs. 11.1 ± 0.5) →Neutral game actions: (0.4 ± 0.1 vs. 0.4 ± 0.1)
Pfeifer et al. (2017) [26]	8 F; 20 ± 2; Highly Trained	N.A.	Power bar; ~1 (100); Before the match and each set	Placebo (decaffeinated gel)	SM + VJ + AG+ RS (30 m) test	→AG: (8.8 ± 0.7 vs. 8.8 ± 0.4)
Zbinden et al. (2018) [27]	10 M; 19 ± 2; Elite	60	Capsule; 5; 60	Placebo (dextrose)	CMJ test	↑CMJ: +12.2% (46.0 ± 3.0 vs. 41.0 ± 5.0)
Filip-Stachnik et al. (2022) [28]	12 F; 20 ± 2; Elite	186	Gum; ~6 (400); 15	Placebo (cellulose)	VS + SM test	↑Attack jump height: +2.6% (47.2 ± 7.3 vs. 46.0 ± 7.9) ↓Block jump height: −0.3% (34.7 ± 6.2 vs. 34.8 ± 6.4) →Attack point: (4.4 ± 4.3 vs. 3.9 ± 3.7); Serve point: (0.3 ± 0.5 vs. 0.4 ± 0.7)
Filip-Stachnik et al. (2022) [29]	14 F; 26 ± 2; Elite	181	Capsule; 6; 60	Placebo (flour)	CMJ test	→CMJ: (37.9 ± 4.6 vs. 37.8 ± 4.3)
Kaszuba et al. (2022) [30]	9 M/3 F; 23 ± 3; Elite	231	Gum; ~3 (300 for M and 200 for F); 60	Placebo (decaffeinated gum)	VS+ CMJ + SJ + AG + ST (30 m) test	↑Attack accuracy: +20.0% (18.0 ± 3.0 vs. 15.0 ± 4.0) →Serve accuracy: (12.0 ± 4.0 vs. 10.0 ± 3.0); Attack accuracy: (23.6 ± 3.9 vs. 22.5 ± 3.6); Attack jump: (61.4 ± 14.9 vs. 62.4 ± 13.9); Block jump: (48.4 ± 10.6 vs. 48.4 ± 11.6); CMJ: (51.2 ± 11.2 vs. 51.0 ± 11.4); SJ: (39.1 ± 7.8 vs. 40.9 ± 9.6); AG: (9.4 ± 0.7 vs. 9.5 ± 0.8)
Nemati et al. (2023) [31]	15 M; 20 ± 1; Highly Trained	N.A.	Capsule; 3 or 6; 60	Placebo (starch)	VST + Sargent’s Jump + AG+ HS + MBTT +PT test	↑Attack point: +44.2% (9.1 ± 2.6 vs. 6.3 ± 2.5); Serve point: +20.9% (10.8 ± 1.7 vs. 8.9 ± 1.6) →AG: (18.4 ± 1.5 vs. 18.5 ± 2.2)
Siquier-Coll et al. (2023) [32]	8 F; 21 ± 4; Highly Trained	100	Powder; 5; 60	Placebo (maltodextrin)	WQ + CMJ + RJ + COD 505 + Yo-Yo test	↑CMJ: +11.4% (34.2 ± 5.6 vs. 30.7 ± 4.4); RJ: +9% (30.3 ± 3.4 vs. 27.8 ± 3.0); HS: +7.2% (348.7 ± 36.4 vs. 325.3 ± 31.5)

→: no change; ↑: increase; ↓: decrease; AG: agility; CMJ: countermovement jump; COD 505: change of direction 505 test; F: female; HS: handgrip strength; ST: sprint test; M: male; MBTT: medicine ball throw test; N.A.: not available; PT: plate tapping test; RJ: repeated jump; RS: repeated sprint; RSE: repeated sprint exercise; SM: simulated match; SJ: squat jump; VJ: vertical jump; VS: spike-ball velocity; VST: volleyball-specific skill test; WQ: wellness questionnaire.

## Data Availability

The original contributions presented in this study are included in the article. Further inquiries can be directed to the corresponding author.
